# MTA2 knockdown suppresses human osteosarcoma metastasis by inhibiting uPA expression

**DOI:** 10.18632/aging.206070

**Published:** 2024-09-06

**Authors:** Chun Tseng, Chien-Min Chen, Yi-Hsien Hsieh, Chia-Yu Lin, Jian-Wen Chen, Pang-Hsuan Hsiao, Yi-Chin Fong, Pei-Han Wang, Pei-Ni Chen, Renn-Chia Lin

**Affiliations:** 1 Graduate Institute of Biomedical Sciences, China Medical University, Taichung, Taiwan; 2 Department of Orthopedic Surgery, China Medical University Hospital, Taichung, Taiwan; 3 Spine Center, China Medical University Hospital, Taichung, Taiwan; 4 Department of Orthopedic Surgery, China Medical University Beigang Hospital, Yunlin, Taiwan; 5 Division of Neurosurgery, Department of Surgery, Changhua Christian Hospital, Changhua, Taiwan; 6 Department of Leisure Industry Management, National Chin-Yi University of Technology, Taichung, Taiwan; 7 Department of Biomedical Sciences National Chung Cheng University, Chiayi, Taiwan; 8 Institute of Medicine, Chung Shan Medical University, Taichung, Taiwan; 9 Department of Sports Medicine, College of Health Care, China Medical University, Taichung, Taiwan; 10 Department of Orthopedics, Chung Shan Medical University Hospital, Taichung, Taiwan; 11 School of Medicine, Chung Shan Medical University, Taichung, Taiwan

**Keywords:** osteosarcoma, MTA2, metastasis, ERK, uPA

## Abstract

The relationship between metastasis-associated protein 2 (MTA2) overexpression and tumor growth and metastasis has been extensively studied in a variety of tumor cells but not in human osteosarcoma cells. This study aims to elucidate the clinical significance, underlying molecular mechanisms, and biological functions of MTA2 in human osteosarcoma *in vitro* and *in vivo*. Our results show that MTA2 was elevated in osteosarcoma cell lines and osteosarcoma tissues and was associated with tumor stage and overall survival of osteosarcoma patients. Knockdown of MTA2 inhibited osteosarcoma cell migration and invasion by reducing the expression of urokinase-type plasminogen activator (uPA). Bioinformatic analysis demonstrated that high levels of uPA in human osteosarcoma tissues correlated positively with MTA2 expression. Furthermore, treatment with recombinant human uPA (Rh-uPA) caused significant restoration of OS cell migration and invasion in MTA2 knockdown osteosarcoma cells. We found that ERK1/2 depletion increased the expression of uPA, facilitating osteosarcoma cell migration and invasion. Finally, MTA2 depletion significantly reduced tumor metastasis and the formation of lung nodules *in vivo*. Overall, our study suggests that MTA2 knockdown suppresses osteosarcoma cell metastasis by decreasing uPA expression via ERK signaling. This finding provides new insight into potential treatment strategies against osteosarcoma metastasis by targeting MTA2.

## INTRODUCTION

Osteosarcoma is a skeletal system malignant tumor that occurs primarily in younger patients. Among adults, osteosarcoma is the third most common tumor after chondrosarcoma and chordoma. The global annual incidence rate of osteosarcoma is 3.4 cases per one million people [1], and the leading cause of death in osteosarcoma is lung metastasis [2]. Osteosarcoma is characterized by the production of calcified osteoid matrix by tumor cells and a high propensity for lung metastasis [3]. Despite recent progress in treatment using a combination of chemotherapy and surgery, the prognosis for OS patients remains poor, and the 5-year survival rate is low [4]. The molecular mechanisms underlying osteosarcoma progression and drug resistance are still not fully understood.

Tumor metastasis, the spread of cells from the primary tumor to other sites to form secondary tumors, is a major factor contributing to cancer-related mortality [5]. Metastasis-associated protein 2 (MTA2), a member of the metastasis-associated transcription regulator family, is a core component of nucleosome remodeling and histone deacetylation complexes [6]. Several studies suggest that MTA2 is highly expressed in human cancers and is directly associated with malignancy, metastasis, drug resistance, and a poor cancer prognosis [7]. Other studies have shown that MTA2 is overexpressed in a variety of cancers, including cervical [8], liver [9], breast [10], and lung [11]. The inhibition of MTA2 expression in human renal cancer cells decreases their invasiveness and metastasis through the miR-133b/MMP9 pathway [12]. Overexpression of MTA2 contributes to the growth, metastasis, and epithelial-mesenchymal transition (EMT) progression of esophageal squamous cell carcinoma through the EIF4E-Twist pathway [13]. Depletion of MTA2 suppresses oral cancer cell metastasis via the p-cofilin-1/LC3-II pathway [14]. Thus, mounting evidence suggests that MTA2 plays a crucial role in tumor progression and affects cancer prognosis. However, its role in osteosarcoma remains unclear and requires further investigation.

Urokinase plasminogen activator (uPA) is a serine protease that catalyzes the conversion of plasminogen to its active form, plasmin. Plasmin then contributes to extracellular matrix (ECM) degradation, a process that is essential to tumor proliferation, metastasis, and angiogenesis [15, 16]. Elevated uPA expression is associated with poorer survival among patients with pancreatectomy [17], hepatocellular carcinoma [18], renal cancer [19] and glioma [20]. There is involvement of uPA expression in metastatic OS cells and in the osteosarcoma microenvironment [21]. Wu et al. found that COX2 knockdown in OS-732 cells significantly inhibited uPA expression and their invasiveness [22]. Therefore, uPA expression plays an important role in osteosarcoma metastasis. However, the relationship between MTA2 and uPA expression and their effects on the metastatic behavior of osteosarcoma are unclear.

The present study aims to determine whether MTA2 is overexpressed in human osteosarcoma tissues and cells and whether MTA2 expression levels correlate with clinical data and overall survival rates of osteosarcoma patients. Using a loss-of-function assay, we assess the effects of MTA2 depletion on osteosarcoma cell metastasis *in vitro* and *in vivo* with the goal of determining whether MTA2 is a potential therapeutic target for osteosarcoma.

## RESULTS

### Abnormal expression of MTA2 in osteosarcoma tissues and cell lines

**Figure 1:**
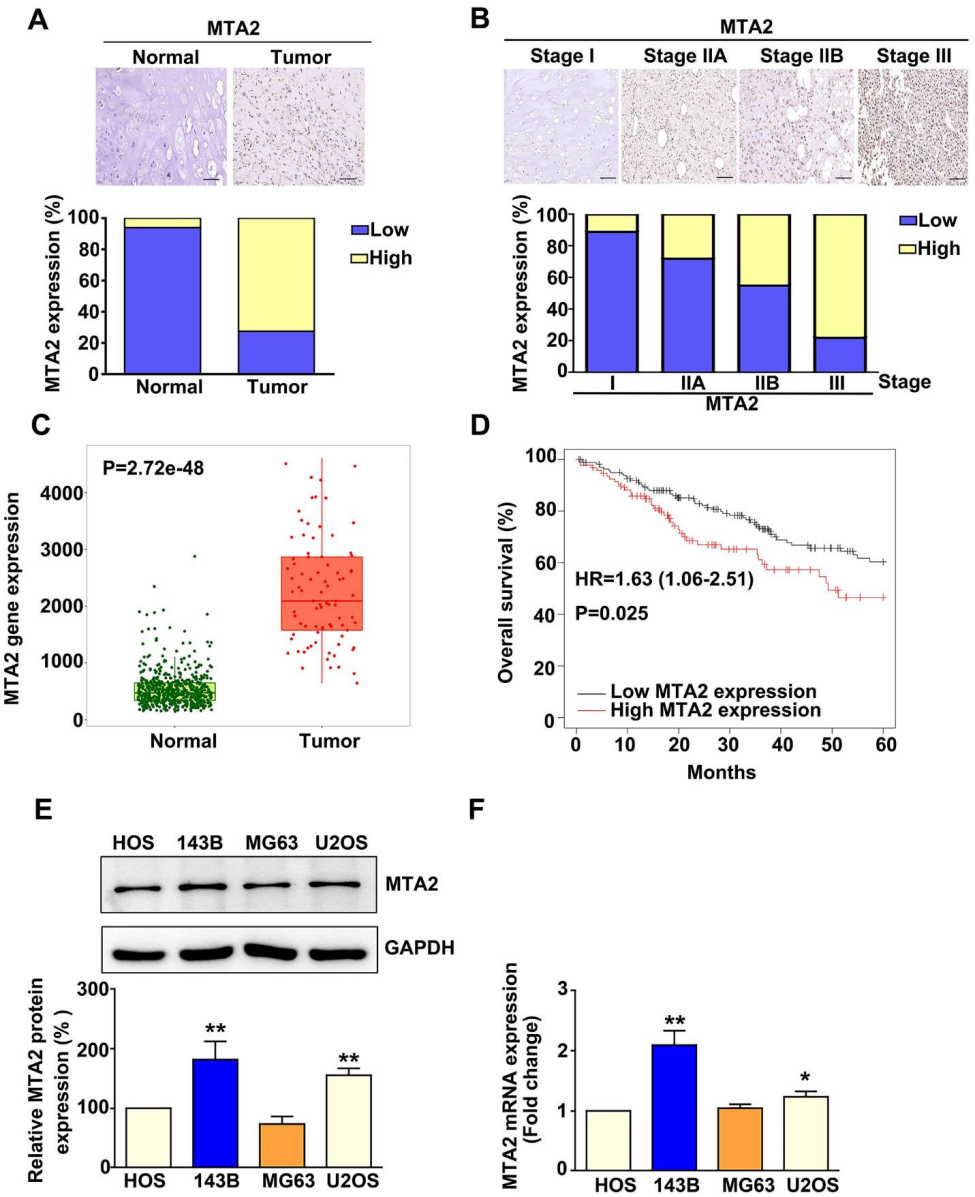
**MTA2 overexpression in human osteosarcoma tissue and osteosarcoma cell lines is associated with poor prognosis in osteosarcoma patients.** (**A**) Representative immunohistochemistry (IHC) results of MTA2 staining in normal and osteosarcoma specimens. Low: low expression of MTA2; High: high expression of MTA2. Scale bar: 50 μm. (**B**) Representative IHC results of MTA2 staining in human osteosarcoma specimens of different tumor stages. (**C**) Comparison of MTA2 expression between normal and tumor tissues from osteosarcoma patients using TNMplot from the TCGA database. (**D**) Kaplan–Meier analysis of overall survival in osteosarcoma patients with high and low MTA2 expression levels. (**E**, **F**) Protein and mRNA expression of MTA2 in 4 human osteosarcoma cell lines, as assessed using western blot and RT-qPCR assays. ** P < 0.01; # P < 0.05.

To examine the clinical significance of MTA2 in human osteosarcoma patients, we analyzed MTA2 expression in a human osteosarcoma tissue array. Compared to normal tissues, MTA2 protein expression was upregulated in osteosarcoma tumor tissue (P < 0.01) (Figure 1A). Furthermore, MTA2 expression was greater in osteosarcoma tissue of stages II (IIA+IIB) and III than in that of stage I (P < 0.01) (Figure 1B). Analysis of data in TCGA using the TNMplot software showed that the MTA2 protein level was significantly higher in osteosarcoma tissue than in normal tissue (Figure 1C, P < 0.001). Notably, Kaplan–Meier analysis demonstrated that higher MTA2 levels correlated with poorer overall survival in human OS patients (Figure 1D; HR, 1.62; P = 0.019). These findings indicate that MTA2 upregulation of may be important in human osteosarcoma progression.

### Effects of MTA2 knockdown on human osteosarcoma cell growth, proliferation, migration, and invasion

**Figure 2:**
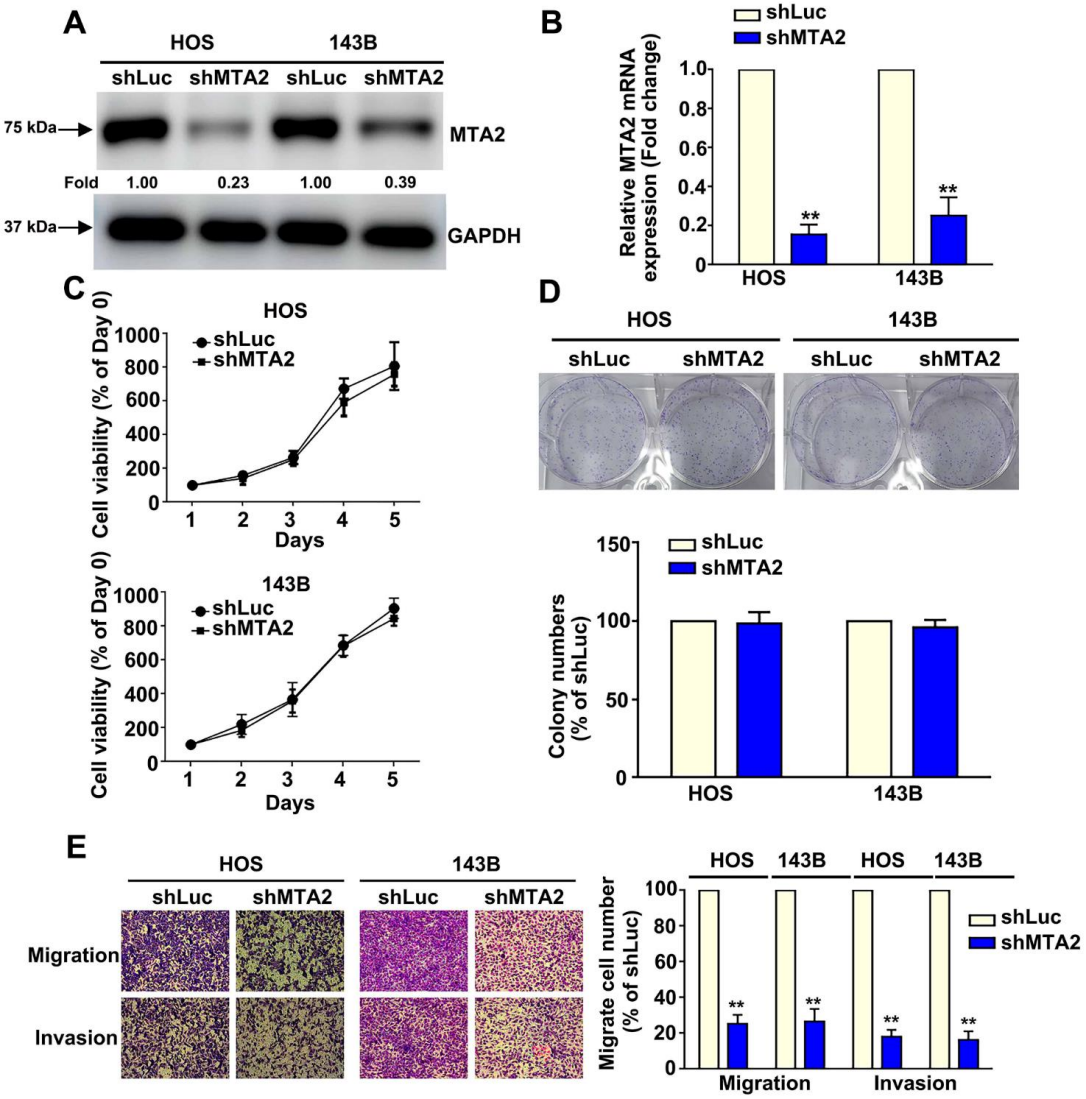
**Knockdown of MTA2 inhibits human osteosarcoma cell migration and invasion.** (**A**, **B**) Stable MTA2 knockdown in HOS and 143B cells was ascertained by western blot and RT-qPCR assay. (**C**) Cell growth ability of shLuc- and shMTA2- osteosarcoma cells by MTT assay. (**D**) Proliferation rates of shLuc- and shMTA2- osteosarcoma cells as assessed by colony formation assay. (**E**) *In vitro* migration and invasion assays to evaluate the effect of MTA2 knockdown on the migratory and invasive abilities of HOS and 143B cells. ** P < 0.01.

To investigate the biological role of MTA2 in human osteosarcoma progression, loss-of-function studies were conducted using HOS and 143B cells (Figure 1E, 1F). Knockdown of MTA2 protein and mRNA expression in human HOS and 143B cells was confirmed by western blot and RT-qPCR assay, respectively (Figure 2A, 2B). Additionally, we explored the effects of MTA2 knockdown on *in vitro* cell growth and proliferation using the MTT and colony formation assays. The results show that MTA2 knockdown did not have a significant influence on the growth or proliferation rate of HOS or 143B cells (Figure 2C, 2D. Notably, MTA2 knockdown significantly reduced the migration and invasion capabilities of both HOS and 143B cells (Figure 2E). Taken together, these findings show that MTA2 inhibition resulted in a decrease in OS metastasis, independent of cell proliferation.

### Inhibition of MTA2 reduced uPA expression and correlation between MTA2 and uPA levels with clinical OS tissues

**Figure 3:**
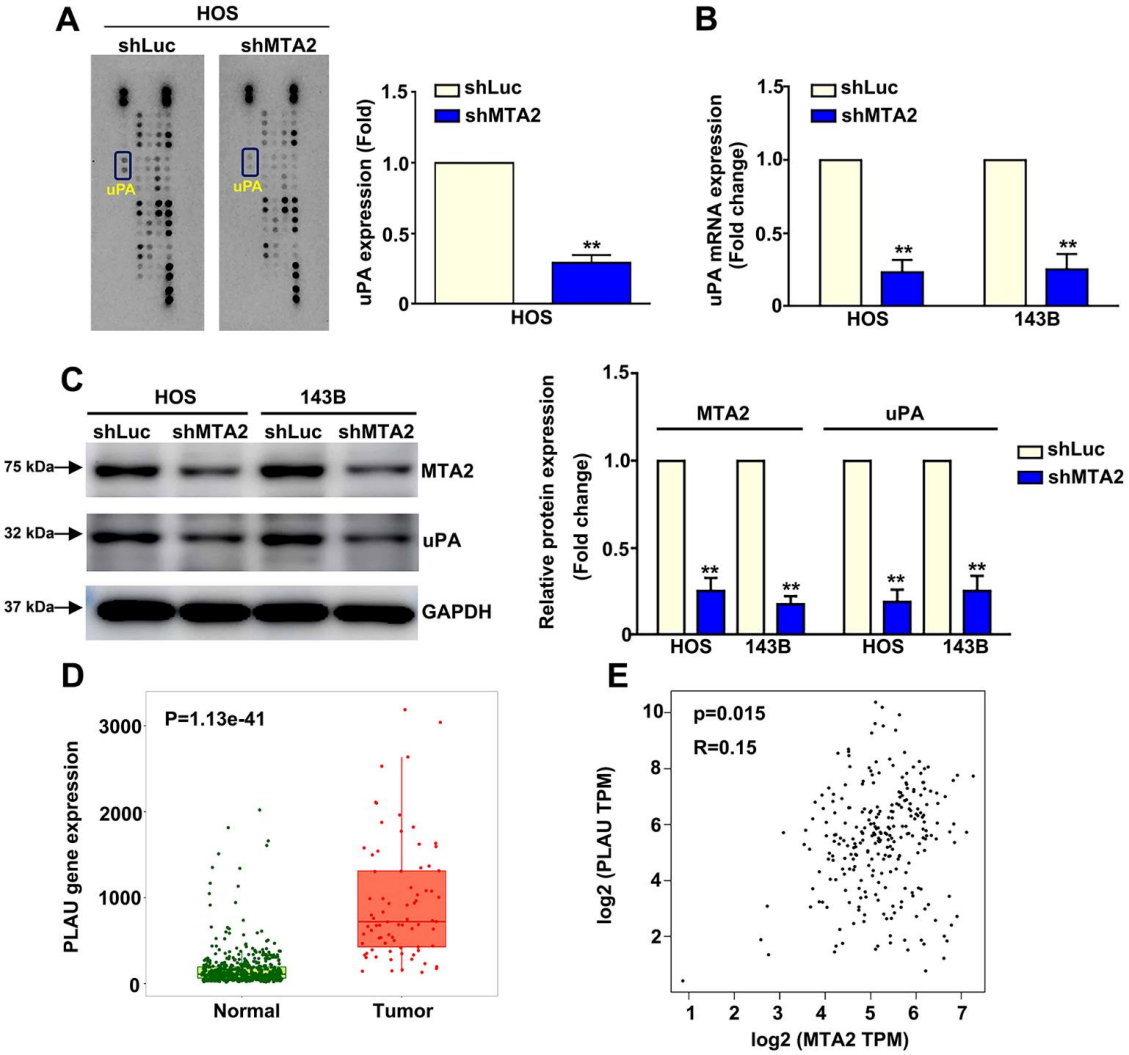
**MTA2 depletion inhibits human osteosarcoma metastasis by decreasing uPA expression.** (**A**) shLuc and shMTA2 osteosarcoma cells were extracted, collected, and detected using a human proteinase array. (**B**, **C**) The mRNA and protein expression of uPA in both osteosarcoma cell lines by RT-qPCR and western blot assay. (**D**) Analysis of MTA2 expression between normal and tumor tissues from osteosarcoma patients using the TNMplot database. (**E**) Correlation analysis between MTA2 and uPA expression in human osteosarcoma tissues using GEPIA software. ** P < 0.01.

Screening of a human proteinase array containing 35 protease proteins to identify MTA2-targeted proteins showed that lower MTA2 expression correlated with lower uPA expression in HOS cells (Figure 3A). This result was supported by RT-qPCR and western blot analysis results showing decreased uPA mRNA and protein expression in MTA2-knockdown-143B and HOS cells compared with shLuc-143B and -HOS cells (Figure 3B, 3C). Using TNMplot software to screen the TCGA database, we observed that uPA expression was elevated in human osteosarcoma tissues compared to that of normal tissues (P = 1.13e^-41^) (Figure 3D). Additionally, we found a positive correlation between uPA and MTA2 expression in OS patient tissues (R = 0.15; P = 0.015) by using the GEPIA database (Figure 3E). Overall, these findings suggest that uPA may be a biomarker for osteosarcoma and that involved in the molecular mechanism underlying of the involvement of MTA2 in human osteosarcoma metastasis.

### Effect of recombinant uPA treatment on cell migration and invasion in human osteosarcoma cells with MTA2 knockdown

**Figure 4:**
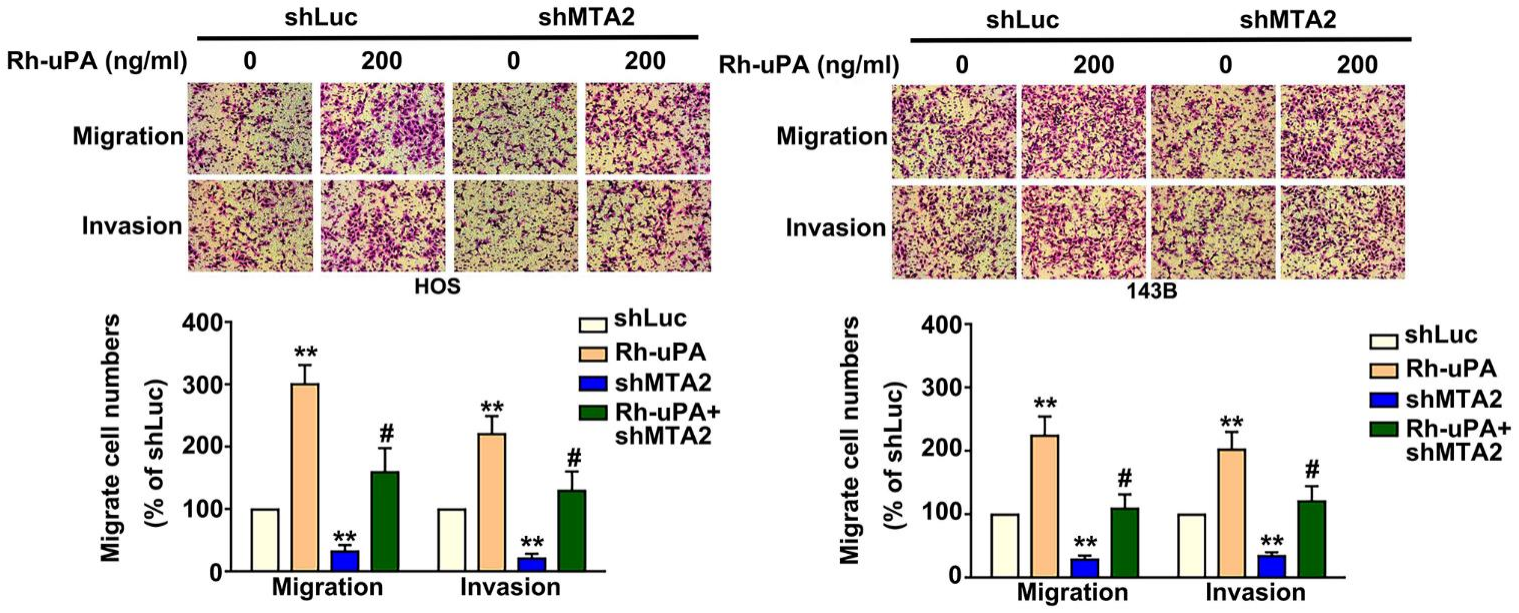
**Effect of uPA treatment on migratory and invasive capabilities in human MTA2-depleted osteosarcoma cells.** Treatment with or without Rh-uPA (200 ng/mL) in shLuc and shMTA2 osteosarcoma cells for 24 h, followed by the assessment of cell migration and invasion ability using the *in vitro* Boyden chamber migration and invasion assay. ** *P < 0.01*, compared with shLuc cells; # *P < 0.05*, compared with shMTA2 cells.

The action of uPA as a proteinase is proven to sequentially degrade the extracellular matrix, thereby triggering tumor cell metastasis [15]. Our results show that treatment with recombinant uPA (Rh-uPA) protein increased osteosarcoma cell migration (HOS cells, 298.5% increase; 143B cells, 234.2% increase) and cell invasive ability (HOS cells, 220.2% increase; 143B cells, 204.1% increase), compared with shLuc-OS cells (Figure 4). Inhibiting MTA2 expression significantly reduced cell migration (shMTA2-HOS cells, 85.2% decrease; 88.8% decrease in shMTA2-143B cells) and invasion (shMTA2-HOS cells, 87.5% decrease; shMTA2-143B cells, 80.2% decrease) (Figure 4). Treatment with Rh-uPA protein restored the osteosarcoma cell migration (shMTA2-HOS cells, 167.3% increase; shMTA2-143B cells, 114.5% increase) and invasion (shMTA2-HOS cells, 141.2% increase; shMTA2-143B cells, 122.5% increase) by MTA2 depletion, compared with shMTA2-OS cells (Figure 4). These results indicate that uPA is involved in the MTA2-induced promotion of osteosarcoma metastasis.

### ERK1/2 is essential for MTA2/uPA-mediated migration and invasion ability of human osteosarcoma cells

**Figure 5:**
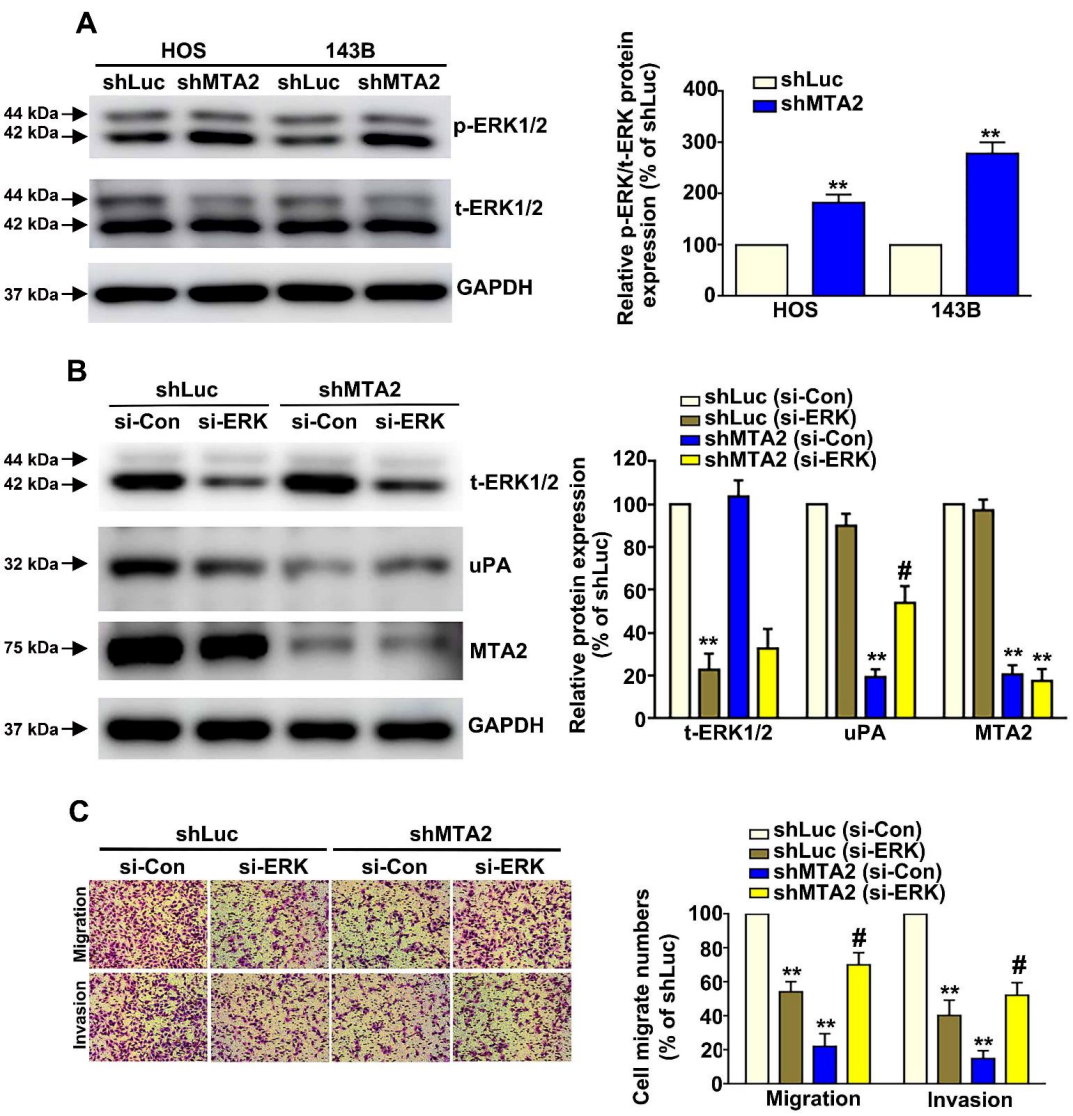
**ERK1/2 is involved in MTA2-mediated regulation of human osteosarcoma cell migration and invasion.** (**A**) The expression of activated ERK1/2 in shLuc and shMTA2 cells by western blot assay. (**B**) Transfection of si-Con or si-ERK in shLuc and shMTA2 osteosarcoma cells for 24 h, followed by the western blot and (**C**) *in vitro* Boyden chamber migration and invasion assay. ** *P* < 0.01, compared with shLuc cells; # *P* < 0.05, compared with shMTA2 cells.

Considering the importance of the ERK1/2 pathway in osteosarcoma metastasis [23], we next focused on the molecular mechanism of ERK1/2 in human osteosarcoma cells. Western blot assay results show that MTA2 knockdown induced ERK1/2 phosphorylation in both osteosarcoma cell lines, with no change in the total ERK1/2 protein level (Figure 5A). Using siRNA-ERK to block endogenous ERK1/2 expression in HOS cells, we observed a decrease in the increased expression of uPA induced by MTA2 depletion (Figure 5B). MTA2 knockdown led to a significant decrease in the migration and invasive ability of HOS cells, whereas silencing ERK reversed the cell migration and invasion inhibited by MTA2 depletion (Figure 5C).

### MTA2 knockdown mitigated osteosarcoma metastasis *in vivo*

**Figure 6:**
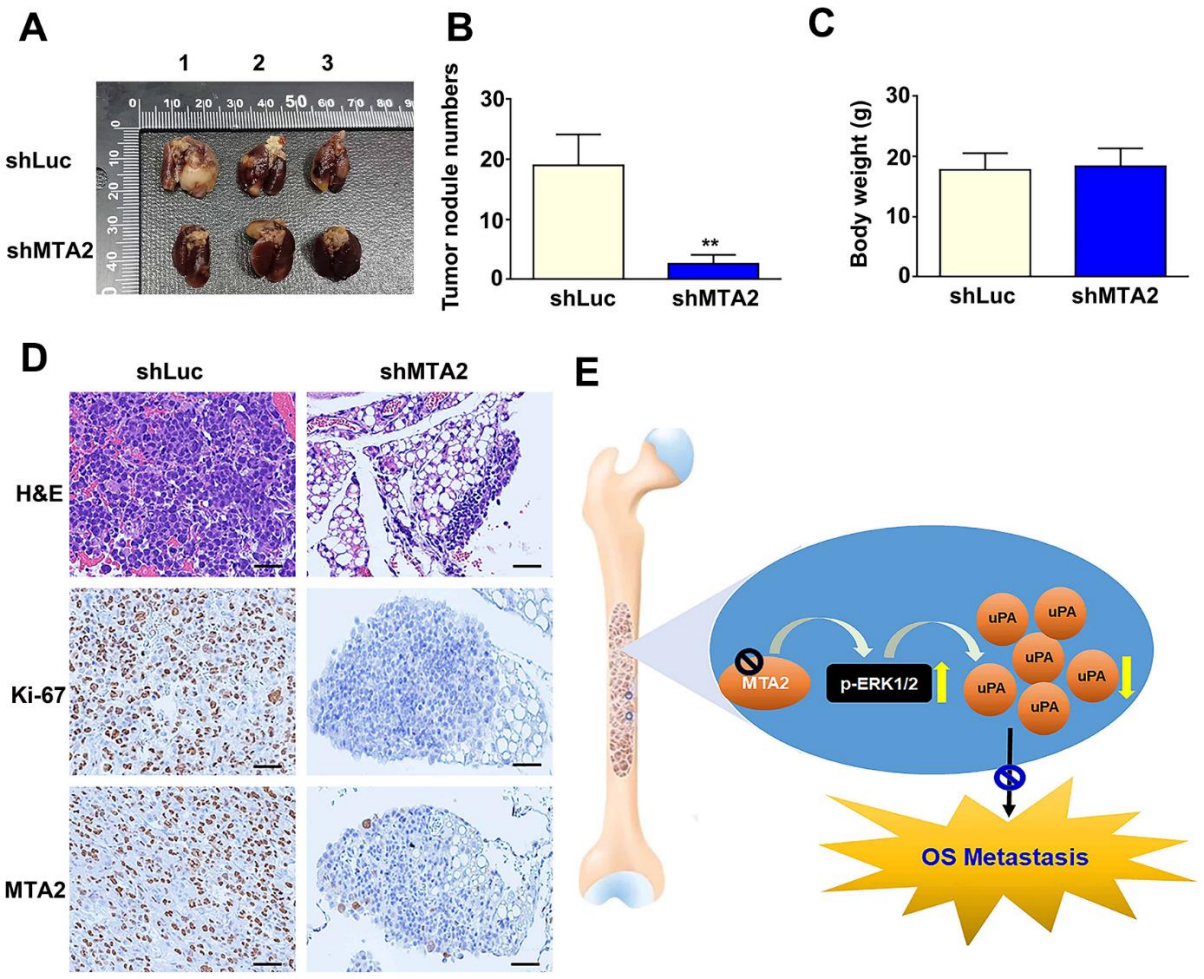
**MTA2 depletion inhibits osteosarcoma metastasis *in vivo*.** (**A**) shLuc- and shMTA2-143B cells (1 × 10^6^ /0.1mL) were injected into the tail vein of C.B17-SCID mice for ten weeks; mice were then sacrificed and the lung tissues removed, shLuc and shMTA2 143B lung tumor excised from C.B17-SCID mice. (**B**) Number of nodules in lung tumors and (**C**) body weight among shLuc and shMTA2 mice. (**D**) H&E staining for tissue morphology and IHC staining for Ki-67 and MTA2 in shLuc and shMTA2 mice. Scale bar: 100 μm. (**E**) Depiction of the role and biological function of the control of uPA expression by MTA2 in human osteosarcoma metastasis.

To determine the effect of MTA2 on the progression of metastasis in osteosarcoma *in vivo*, we introduced MTA2-depleted 143B cells with lentiviral infection into mice. We observed that MTA2 depletion led to a reduction in lung metastasis (Figure 6A) and a decrease in lung nodules compared with luciferase knockdown (shLuc) control mice (Figure 6B). Importantly, there was no noticeable difference in body weight between the shLuc and shMTA2 mice (Figure 6C). Immunohistochemical assay revealed that significantly decreased the Ki-67 and MTA2 expression in MTA2 depletion mice, compared with luciferase knockdown (shLuc) control mice (Figure 6D). Taken together, our findings suggest that MTA2 plays a critical role in human osteosarcoma metastasis.

## DISCUSSION

While osteosarcoma comprises malignant osteogenic cells, no well-defined molecular markers are presently known to effectively predict osteosarcoma progression. An increasing number of studies suggest the involvement of aberrant MTA2 expression in tumor proliferation, metastasis, and chemotherapeutic agent resistance and indicate that MTA2 expression levels correlate with tumor characteristics such as size, grade, and lymph node metastasis in a variety of cancers [24, 25]. Our previously published results show that MTA2 serves as a prognostic factor for human cervical cancer [8], renal cancer [12], and hepatocellular carcinoma [9]; however, less is known about the clinical significance of MTA2 in human osteosarcoma patients. The present study is the first to report the overexpression of MTA2 in osteosarcoma tissues, and its correlation with tumor stage and survival rates of osteosarcoma patients. Moreover, our *in vitro* study results revealed high levels of MTA2 expression in osteosarcoma cells. Therefore, our findings indicate that MTA2 overexpression promotes osteosarcoma progression.

We observed elevated levels of MTA2 expression in 4 osteosarcoma cell lines. MTA2 knockdown in HOS and 143B cells decreased cell migration and invasion, independent of cell growth. *In vivo* animal experiments revealed that MTA2 depletion reduced the metastatic potential of osteosarcoma cells, as indicated by a decrease in the number of metastatic nodules. These results are consistent with previous reports of MTA2 involvement in the metastasis of other cancers, including cervical cancer [26], esophageal squamous cell carcinoma [13], and nasopharyngeal carcinoma [27]. These findings suggest that MTA2 is a key factor in the metastatic progression of osteosarcoma.

To further understand the molecular mechanism underlying the role of MTA2 in osteosarcoma metastasis, we performed human proteinase analysis in MTA2-depleted osteosarcoma cells. UPA protein expression was significantly downregulated in MTA2-depleted cells, and uPA overexpression restored the migration and invasive ability of MTA2-depleted cells. Accumulating evidence indicates that excess uPA degrades the extracellular matrix, which is associated with the metastatic process in a variety of solid tumors, including breast, uterine cervix, pancreas, ovary, and glioma [28, 29]. Endo-Munoz et al. reported that the conversion of malignant osteosarcoma cells to a metastatic state is characterized by activation of the uPA/uPAR system [21]. We found that uPA expression was higher in osteosarcoma tissues than in normal tissue and correlates positively with MTA2 expression, suggested that uPA may be useful as a predictive or prognostic biomarkers for osteosarcoma progression. Ghasemi et al. discovered that the RhoA/ROCK signaling pathway affects the migration and invasion of ovarian cancer cells by modulating uPA expression [30]. In breast cancer cells, the inhibition of TIPE3 expression significantly decreased uPA expression through activation of the AKT/NF-κB pathway [31]. Silencing of uPA expression reduced MDA-MB-231cells migratory, invasive, and adhesive ability and modulated the EMT by down-regulating Oct-4 expression [32]. Our study findings are consistent with these other studies, suggesting that MTA2 depletion decreased the metastatic capacity of osteosarcoma cells by inhibiting uPA expression.

The extracellular signal-regulated kinase (ERK)1/2 pathway is involved in the regulation of a variety of cancers and is thus of interest for potential therapeutic development. Most of this research is focused on inhibiting ERK to reduce cell migration and invasion in tumor cells [33]. For instance, in gallbladder cancer, EMP3 inhibition activates the ERK1/2 pathway, leading to the inhibition of cell proliferation and metastasis [34]. In a study of osteosarcoma, LCN2 depletion promoted cell migration and invasion by modulating the MEK-ERK pathway [35], and the MEK inhibitor U0126 significantly decreased osteosarcoma tumor invasion and metastasis *in vivo* [36]. However, ERK activation is not always associated with cell survival and proliferation. Recent studies report that ERK activation is associated with senescence and cell death signaling [37]. Thus, the MAPK/ERK signaling pathway is a double-edged sword in tumor progression.

To investigate whether ERK1/2 signaling acts downstream of MTA2, we first examined the role of ERK1/2 activation in MTA2-depleted osteosarcoma cells. Our result revealed that MTA2 inhibited osteosarcoma metastasis via activation of ERK1/2-inhibited uPA signaling. The possible transcription and translation mechanisms underlying ERK-mediated metastasis of osteosarcoma cells inhibited by MTA2 are under investigation. We also must consider whether the uPA receptor (uPAR) is regulated by MTA2; accordingly, further studies will investigate the regulatory roles of the uPA/uPAR/ERK signaling pathway in osteosarcoma progression.

## CONCLUSIONS

In summary, MTA2 depletion decreased osteosarcoma cell migration and invasion by inducing the ERK1/2 phosphorylation-mediated uPA pathway (Figure 6E). This finding suggests that the MTA2/uPA axis may be a potential target for therapeutic agents against osteosarcoma.

## MATERIALS AND METHODS

### Antibodies, chemicals and reagent

Antibodies against MTA2, uPA, and GAPDH were purchased from Santa Cruz Biotechnology (Santa Cruz, CA, USA). siRNA-ERK was designed and synthesized by GenePharma (Shanghai, China). Human osteosarcoma tissue array (OS802c) was purchased from US Biomax (Derwood, MD, USA). The human protease array kit (ARY021B) was purchased from R&D Systems, Inc. (Minneapolis, MN, USA). Recombination-human uPA was from SinoBiological (Beijing, China).

### Cell lines and culture condition

The HOS (60308), 143B (60439), MG63 (60279) and U2OS (60187) human osteosarcoma cell line were obtained from the Bioresource Collection and Research Center (BCRC) (Hsinchu, Taiwan). These osteosarcoma cells were maintained with MEM medium containing 10% fetal bovine serum (FBS), 0.1 mM non-essential amino acids (NEAA), 1.0 mM sodium pyruvate and 100 U/mL penicillin–streptomycin (Invitrogen Life Technologies, Carlsbad, CA, USA). The cell cultures condition was maintained in a humidified incubator under at 37° C with 5% CO_2_.

### Stable expressing shRNA-MTA2 osteosarcoma cell line

The shMTA2 (clone ID: TRCN0000232200) was purchased from the RNA Technology Platform and Gene Manipulation Core Facility (RNAi core) of the National Core Facility (Taipei, Taiwan). The MTA2 targeting sequences are 5’-AGGGAGTGAGGAGTGAATTAA-3’, the pLKO.1-Luc as scrambled control. For MTA2 knockdown, HEK-293T cells were co-transfected with pCMV∆R8.91, pMD.G, and the pLKO.1-puro-expressing vector using Lipofectamine 3000 transfection reagent and incubated for 6 h. After changing the medium, the cells were incubated for 48 h and the viral supernatant collected and filtered through a 0.45-μm filter. Human osteosarcoma cells were then infected with the shLuc and shMTA2 for 24 h in the presence of polybrene (8 μg/mL). MTA2 expression in osteosarcoma cells was assessed by western blot analysis and RT-qPCR assay.

### Cell proliferation assays

Colony formation assay was performed to assess the cell proliferative rate. The shLuc- and shMTA2-osteosarcoma cells (1×10^3^/well) were seeded in 6-well plate and incubated for 7 days, then stained with crystal violet reagent (1:20) for 30 mins. The number of colonies in shLuc- and shMTA2-osteosarcoma cells was counted under the microscope. Colony formation efficiency (%) = (numbers of shMTA2 cell colonies/ number of shLuc cell colonies) **×** 100%.

### Boyden chamber cell migration and invasion assay

To assess the effects of MTA2 knockdown and uPA overexpression on cell migration and invasion in human osteosarcoma cells *in vitro*, we used a Boyden chamber migration assay with or without Matrigel coating. The MTA2 knockdown and uPA-overexpressing OS cells (4 × 10^5^/mL) were seeded into the upper part of the Boyden chamber (Neuro Probe, Cabin John, MD, USA). To the lower chamber was added culture medium with 20% FBS. The cells were incubated at 37° C for 18 h (migration assay) or 24 h (invasion assay). The migrated cells were fixed and stained with Giemsa reagent (1:20) for 30 mins and then counted in 5 fields of vision under a light microscope.

### Human protease array

Human Protease Array contained the 35 different proteinase proteins. The shLuc and shMTA2-HOS cells were lysed with Lysis Buffer 17 supplemented with 10 μg/mL Aprotinin/Leupeptin/10 μg/mL and Pepstatin A 35 μg/mL, rock the lysates gently at 4° C for 20 minutes, then incubated the membranes and blocking for 1 h. Next, Protease Detection Antibody was added to each sample for 1 h at room temperature. Finally, the 1X Streptavidin-HRP reagent was added into membrane and detected the specific protein expression.

### Recombinant uPA protein assay

Treatment with or without 200 ng/ml Recombinant uPA (Rh-uPA) protein in shLuc and shMTA2-osteosarcoma cells for 24 h was done, then measured the cell migration and invasion abilities by *in vitro* cell migration and invasion assay.

### RT-qPCR assay of MTA2 and uPA mRNA expression

Total cellular RNA was extracted using TRIzol reagent (Thermo Fisher Scientific, Waltham, MA, USA) according to the manufacturer's protocol. The GoScript reverse transcription mix kit (Promega, Madison, WI, USA) was used to reverse transcribe the RNA (1 μg) into cDNA. Specific cDNAs were amplified using the GoTaq qPCR Master Mix reagent (Thermo Fisher Scientific) using specific PCR primers for MTA2, uPA, and GAPDH with the following sequences: MTA2-Forward, 5’- TGTACCGGGTGGGAGATTAC-3’; MTA2-Reverse, 5’-TGAGGCTACTAG AAATGTCCCTG-3’; uPA-Forward, 5’-CCGCTTTCTTGCTGGTTGTC-3’; uPA-Reverse, 5’- TATTGTCGTTCGCCCTGGTG-3’; GAPDH-Forward, 5’-CATCATCCCTGCCTCTACTG -3’; GAPDH-Reverse, 5’-GCC TGCTTCACCACCTTC-3’. These primer sequences were purchased from Mission Biotech company (Taipei). These gene expression was assessed by the 2^−ΔΔCT^ relative quantitative method using GAPDH as the reference control.

### Western blotting

After cell lysis in NETN lysis buffer, samples of total protein (20 μg) were electrophoresed for 1 h and transferred to a PVDF membrane for 1 h. The membrane was incubated in blocking buffer for 10 min, followed by incubation with primary antibody against MTA2 (1:1000), p-ERK1/2 (1:1000), t-ERK1/2 (1:1000), uPA (1:1000), or GAPDH (1:10000) at 4° C overnight. The membrane was treated with chemiluminescent substrate to quantify protein expression levels.

### Data collection

The Cancer Genome Atlas (TCGA) and TARGET databases in TNMplot (accessible at https://tnmplot.com/analysis/; accessed June 1, 2023) provided us with MTA2 and uPA mRNA expression data and clinical data from 1109 normal samples and 4608 OS tumor samples. The overall survival of human OS patients (n=259) was determined using the Kaplan–Meier plotter database (accessed at https://kmplot.com/analysis/, accessed on 13 September 2023). The correlation between MTA2 and uPA expression was determined using data in the GEPIA database (accessible at http://gepia.cancer-pku.cn/, accessed on 2017).

### *In vivo* lung metastasis mouse model

The *in vivo* metastasis assay was performed as published [38]. The animal research was approved by the Animal Ethics Committee of Chung Shan Medical University (IACUC number: 2771). Experimental procedures followed the Guidelines of Animal Use and Care of the Chung Shan Medical University. The shLuc- or shMTA2-143B cells (1 × 10^6^/0.1 mL) were injected into the tail veins of five weeks old male C.B17-SCID mice (n = 5 mice per group) for 2 months. After ten weeks, the mice were weighed and sacrificed, and the lung tissues were removed. Lung tissue sections were fixed, deparaffinized, and rehydrated. The expression of Ki-67 and MTA2 in the lung tissue sections was determined using immunohistochemical analysis.

### Statistical analysis

The statistical analysis was conducted using GraphPad Prism 6 (GraphPad Inc, San Diego, CA, USA). Student’s t-test or one-way analysis of variance (ANOVA) was exerted to evaluate the prominent distinctions between groups. P < 0.05 was considered a significant difference.
